# Febrile Pancytopenia and Hemophagocytosis From Disseminated Histoplasmosis in HIV/AIDS Patients: Two Cases and a Review of Combined Antifungal and Steroid Therapy

**DOI:** 10.1155/crdi/2623694

**Published:** 2025-04-17

**Authors:** Chinelo Animalu, Nupur Singh, Kenneth Cory Guice, Kase Maner

**Affiliations:** ^1^Division of Infectious Diseases, College of Medicine, University of Tennessee Health Science Center, Memphis, Tennessee, USA; ^2^College of Medicine, University of Tennessee Health Science Center, Memphis, Tennessee, USA; ^3^Department of Pulmonology, University of Alabama, Birmingham, Alabama, USA

## Abstract

Hemophagocytosis is a clinical condition characterized by the engulfment of bone marrow cellular elements, including erythrocytes, leukocytes, platelets, and their precursors, by activated macrophages. It has been associated with several infectious organisms, including the Epstein–Barr virus (EBV) and histoplasmosis. Human immunodeficiency virus (HIV) has been known to trigger hemophagocytosis in the presence or absence of other infections. Disseminated histoplasmosis is a common opportunistic infection in advanced patients with acquired immunodeficiency syndrome (AIDS) in endemic areas; however, the best treatment for histoplasmosis associated with hemophagocytosis is uncertain. This article presents two cases of patients with AIDS secondary to uncontrolled HIV who were admitted with fever, malaise, low CD4 + counts, and a history of noncompliance with antiretroviral therapy (ART). Both patients had pancytopenia, markedly elevated serum ferritin, and elevated liver transaminases. The diagnosis of histoplasmosis was confirmed by positive fungal blood cultures, buffy coat smears showing intracellular fungal organisms, and positive urine *Histoplasma* antigen. Bone marrow biopsies revealed *Histoplasma capsulatum* (*H. capsulatum*) in Grocott methenamine silver (GMS) stains and fungal cultures, histiocytes with intracellular red blood cells, and precursors of granulocytes, consistent with hemophagocytosis. Both patients received amphotericin B but remained febrile and pancytopenic, eventually requiring corticosteroid therapy. We present our experience with these patients and discuss the management of hemophagocytosis in patients with AIDS with disseminated histoplasmosis. We also completed a literature review and created a list of all known cases of disseminated histoplasmosis complicated by HIV/AIDS and hemophagocytosis and listed previous treatments.

## 1. Introduction

Disseminated histoplasmosis is a common opportunistic infection in patients with acquired immunodeficiency syndrome (AIDS) in endemic areas of the Ohio and Mississippi River valleys, also known as the “Histo Belt” [1]. Hemophagocytosis is a potentially fatal complication of disseminated histoplasmosis in patients with AIDS. Given that the three disease entities involved including AIDS, disseminated histoplasmosis, and hemophagocytosis can all present with nonspecific symptoms and febrile pancytopenia, this can lead to delayed diagnosis and subsequent poorer outcomes. Due to the rarity of this combination, the best treatment for histoplasmosis complicated by hemophagocytosis remains uncertain and can vary from patient to patient.

Here, we present two cases of patients with disseminated histoplasmosis who presented persistent febrile pancytopenia, found to be hemophagocytosis. Both patients received amphotericin B and underwent a bone marrow biopsy to establish a diagnosis of hemophagocytosis. The addition of corticosteroids resulted in the resolution of fever and improved pancytopenia.

## 2. Case Reports

### 2.1. Case 1

A 24-year-old African American man with uncontrolled human immunodeficiency virus (HIV)/AIDS diagnosed 2 years prior to admission presented to the hospital with complaints of fever, night sweats, loss of appetite, profound weakness, and 40-pound weight loss. Vital signs showed a blood pressure of 94/58 mm Hg, a pulse of 103 per minute, a temperature of 102.5°F, and a respiratory rate of 24 per minute. On physical examination, he showed evidence of weight loss and mild splenomegaly. No skin lesions were noted; therefore, there was a low suspicion of cutaneous infection. Laboratory studies were significant for hemoglobin of 6.1 g/dL, white blood cell count of 1.700 cu/mm, platelet count of 73,000 cu/mm, elevated transaminases (AST of 312 U/L and ALT of 187 U/L), elevated alkaline phosphatase of 202 U/L, elevated lactate dehydrogenase of 5206 U/L, high ferritin of 48,900 ng/mL, fibrinogen of 100 mg/dL, a CD4^+^ count of two per microliter, and a HIV viral load of 1,395,190 copies/mL. Abdominal ultrasound revealed moderate splenomegaly (13.5 cm).

**Figure 1:**
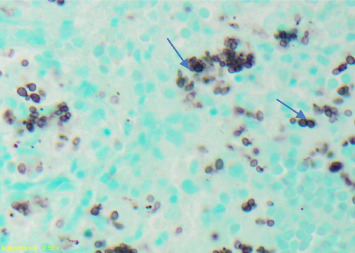
Bone marrow biopsy showing *Histoplasma capsulatum* (blue arrows) on precursors on Grocott's methenamine silver stain (100x magnification).

**Figure 2:**
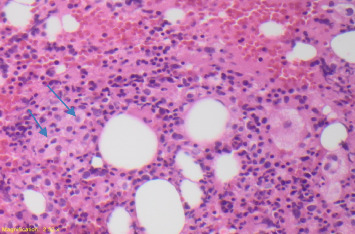
Bone marrow aspirate showing histiocytes with intracellular red cell and granulocyte precursors (blue arrows) on hematoxylin and eosin stain (200x magnification).

Empiric piperacillin–tazobactam, azithromycin, vancomycin, and trimethoprim–sulfamethoxazole were started; however, the patient remained febrile. Given the suspicion of possible invasive fungal infection, amphotericin B was initiated empirically. The fungal blood culture and the serum buffy coat smear subsequently returned positive for *H. capsulatum*. Despite being on amphotericin B, the patient remained febrile. The hematology and oncology team was then consulted due to persistent fever and marked pancytopenia, and a bone marrow biopsy was performed. The bone marrow Grocott methenamine silver (GMS) stain demonstrated numerous fungal organisms consistent with *Histoplasma capsulatum (H. capsulatum*) with diffuse marrow involvement (Figure 1). The fungal culture was positive for *H. capsulatum*. Hematoxylin and eosin (HE) staining of the bone marrow aspirate showed lymphohemophagocytic abnormalities consistent with hemophagocytosis (Figure 2).

On the recommendation of the hematology and oncology team, intravenous dexamethasone 6 mg/6 h was started on hospital Day 4 for HLH with gradual resolution of fever and improvement of pancytopenia. Subsequently, dexamethasone was reduced to an oral regimen of 10 mg daily for Weeks 1 and 2, 5 mg daily for Weeks 3 and 4, 2.5 mg daily for Weeks 5 and 6, and 1.25 mg for Week 7. Amphotericin B was changed to oral itraconazole 200 mg twice daily after 10 days. The patient was discharged with oral itraconazole, prophylactic atovaquone, and azithromycin. He started antiretroviral therapy (ART) consisting of dolutegravir (Tivicay) and emtricitabine/tenofovir (Truvada) in the outpatient clinic 3 weeks after discharge. At 1 year of follow-up, he was well with a normal complete blood count, a CD4^+^ count of 6 μL, and a HIV viral load of 435,580 copies/mL. He continued to receive 200 mg every 12 h of oral itraconazole suppression; weekly 1200 mg azithromycin and 3 times weekly oral trimethoprim–sulfamethoxazole 160 mg were continued for prophylaxis against *Mycobacterium avium* complex and *Pneumocystis jirovecii*, respectively, until the CD4^+^ count was greater than 200 mL.

### 2.2. Case 2

A 27-year-old African American man with newly diagnosed HIV 1 month earlier presented to the emergency department (ED) with complaints of chronic diarrhea, weakness with inability to move, odynophagia, and dysphagia. He denied chest pain or new skin lesions. He was on an ART regimen consisting of emtricitabine/tenofovir (Truvada), darunavir (Prezista), and ritonavir (Norvir). His temperature was measured at 101.6°F in the emergency room. The laboratory findings of admission were significant for pancytopenia with hemoglobin of 5.4 g/dL, white blood cell count of 2.8 cu/mm, and platelet count of 33,000 cu/mm. Other notable values include serum ferritin of 68,000 ng/mL, triglycerides of 136 mmol per liter, CD4^+^ count of 34 μL, and an HIV viral load of 560 copies/mL.

The evaluation of the stool for the infectious etiology of diarrhea was negative. Empiric antibiotics with vancomycin and cefepime were initiated. Blood cultures were negative. Fungal blood cultures had no growth after 2 weeks and the fast blood acid stain was negative. Two acid-fast sputum smears were negative. Abdominal CT (CAT scan) imaging revealed multiple necrotic abdominal lymph nodes. Esophagogastroduodenoscopy (EGD) and colonoscopy were performed. Pertinent findings were multiple superficial ulcers in the retrosigmoid colon, esophagus, and duodenum that, upon biopsy and staining with periodic acid–Schiff–light green (PAS-LG) stain, were confirmed as *Histoplasmosis capsulatum*. Intravenous amphotericin B was initiated with persistent fever and pancytopenia requiring consultation of the hematology and oncology team for further evaluation. A bone marrow biopsy was performed, and the histopathological findings showed a hypercellular marrow and aggregates of histiocytic infiltrates consistent with hemophagocytosis. A histological stain of the bone marrow specimen with GMS stain was also positive for histoplasmosis. After further discussion with the hematology team, a trial of intravenous dexamethasone was added at a dose of 4 mg/6 h with subsequent resolution of the fever after 48 h. Antibiotics were discontinued. Intravenous amphotericin B was later transitioned to 200 mg itraconazole solution twice daily for disseminated histoplasmosis. The patient was discharged on a prolonged course of oral itraconazole 200 mg twice daily and a tapering course of dexamethasone 15 mg orally daily for 2 weeks. Following his discharge, he regularly followed up at the HIV clinic. On his 1-year visit, his CD4 count was 472 μL with a HIV viral load of < 20 copies/mL. He remained on the regimen of Truvada, Prezista, and Norvir.

## 3. Discussion

*H. capsulatum* is a dimorphic fungus that can grow both as a mold in nature or in the culture at room temperature but becomes a small yeast cell at 37°C. Infection occurs through inhalation of spores produced by the yeast form of the fungus that is present in soil or dust contaminated with bird or bat droppings [2]. Infection with histoplasmosis can range from mild asymptomatic infection to severe infection with invasion of multiple systems in the body including the liver, spleen, lungs, bone marrow, brain, and GI tract., appropriately termed as disseminated histoplasmosis. Pulmonary manifestations are often the initial manifestation of disseminated histoplasmosis in HIV patients, commonly presenting with cough, dyspnea, expectoration, and hemoptysis [3]. Fungal infections remain a major cause of opportunistic infection and mortality in patients with AIDS, and reducing the burden of disseminated histoplasmosis is the focus of several efforts [4]. Cutaneous infection can also appear as part of disseminated histoplasmosis presentation. Though various presentations are possible, the most common include skin-colored papules or nodules occasionally with central umbilication, crusting, and drainage [5]. Other rare cutaneous presentations include chancriform lesions and painful ulcers involving the oral mucosa, commonly found in patients with AIDS [6, 7]. If there is high suspicion, a full skin exam must be performed with cultures and skin biopsy of any lesions present. The two patients described here did not have any cutaneous lesions, so no skin biopsy was performed.

Infection in an immunosuppressed host, such as uncontrolled HIV infection, is usually severe with the involvement of multiple systems and is usually life-threatening if not treated promptly. Other triggers of immunodeficiency include various hematologic malignancies, rheumatologic conditions (e.g., systemic lupus erythematosus and rheumatoid arthritis), and organ transplantation [8–11]. Disseminated histoplasmosis can manifest with various nonspecific laboratory and clinical abnormalities with the most common involving fever (95%–96%), splenomegaly (69%–89%), more than cytopenia (92%), high ferritin (94%), hemophagocytosis (82%), liver abnormalities (85%–90%), and others [12–14]. Incidentally, these same symptoms are seen in patients with hemophagocytosis, making the diagnosis incredibly challenging. The diagnosis of disseminated histoplasmosis should be highly considered in patients living in endemic areas, with urine antigen testing being the most common route of diagnosis. Bone marrow biopsy can often show *H. capsulatum* in culture 78% of the time [15].

Even after a prompt diagnosis of disseminated histoplasmosis in patients with febrile pancytopenia and hemophagocytosis, there is no guarantee that antifungal therapy alone will work to reduce symptoms. Coadministration of corticosteroid therapy and ART may be necessary when hemophagocytosis presents as an additional complication. For our patients, we had minimal response with liposomal amphotericin B alone, prompting the addition of dexamethasone with resolution of symptoms and laboratory abnormalities. For hemophagocytosis associated with disseminated histoplasmosis in patients with HIV, therapy should include antifungal agents, preferably liposomal amphotericin B, initially for induction and later for transition to oral itraconazole. The use of high-dose dexamethasone can be added if there is no response to antifungal therapy alone. This trifecta of treatment: antifungal, antiretroviral, and steroids, has had some success in documented cases before and is further supported by positive findings in our patients [16]. In severe cases, chemotherapy with regimens containing etoposides or cyclosporine with a corticosteroid may be required to control this hyperinflammatory syndrome. Refractory hemophagocytosis may ultimately require hematopoietic cell transplantation (HCT) or biologics (e.g., emapalumab and alemtuzumab) [17–19]. However, the treatment of disseminated histoplasmosis with hemophagocytosis in HIV/AIDS patients remains variable. A review of case reports worldwide shows that there is no consistent regimen for this condition, as each case was treated differently according to the clinical status of the patient (Table 1).

## 4. Conclusions

These cases highlight the importance of considering hemophagocytosis in patients with HIV or other immunosuppressive conditions who live in regions endemic to certain diseases such as *H. capsulatum*, anaplasma, ehrlichia, or other infectious etiologies and in patients who remain pancytopenic or do not respond to treatment of the underlying infection. An examination of the bone marrow may be necessary to help establish the presence of underlying hemophagocytosis. Our patients demonstrated positive responses to antifungal treatment with the addition of corticosteroid therapy, highlighting the need for better mainstay pharmacological management. There are still no randomized clinical trials to support the definitive treatment of this condition secondary to infectious etiologies; however, treatment of underlying infectious etiologies is paramount to improve survival.

## Figures and Tables

**Table 1:** All cases of histoplasmosis-associated hemophagocytosis in HIV patients.

Country, year	Age	Sex	Race	Number of patients	Inpatient treatment regimen + outpatient (O)	Clinical outcome	References
Mexico, 1993	37, 49, 36	M	H/L	3	AB, fluconazole	S (2), D (1)	Majluf-Cruz et al. [20]
United States of America 1995	29, others unknown	1 M, others unknown	N/A	6	AB, AB + IVIG	S (3), D (3)	Koduri et al. [21]
France, 1997	50	M	B	1	NA	N/A	Chemlal et al. [22]
India, 2000	40	M	A	1	NA	D	Kumar et al. [23]
Spain, 2007	33	M	H/L	1	N/A	D	Gil-Brusola et al. [24]
Puerto Rico, 2007	43	M	H/L	1	ABLC + itra (O) + HAART (O)	S	Guiot et al. [25]
United States of America, 2007	61	M	H/L	1	Lip AB + itra (O)	S	Sanchez et al. [26]
France, 2009	33	M	N/A	1	ABD + itra + IVIG + HAART	S	De Lavaissière et al. [27]
United Kingdom, 2011	25	M	N/A	1	Antifungal treatment + HAART	D	Vaid and Patel [28]
India, 2012	38	F	A	1	Ketoconazole	S	Chandra et al. [29]
United States of America, 2012	28	M	B	1	Voriconazole + HAART	D	Telfer and Gulati [30]
United States of America, 2014	25	M	H/L	1	Dex + antifungal treatment + HAART	S	Huang [31]
United States of America/Mexico/El Salvador, 2015	31, 53, 33, 28, 44, 52 (2), 32, 51	M (7), F (2)	H/L, W	9	Lip AB + itraconazole + prednisone, Lip AB, Lip AB + itra, Lip AB + itra, Lip AB + fluconazole, Lip AB + itra + steroids + IVIG, Lip AB + itra + IVIG, Lip AB + steroids	S (5), D (4)	Townsend et al. [32]
United States of America, 2015	32	M	H/L	1	Lip AB + itra + Dex + etoposide + HAART (O)	S	Castelli et al. [33]
United States of America, 2015	42	F	N/A	1	Lip AB + itra (O) + HAART (O)	S	Subedee and Van Sickels [34]
Colombia, 2016	33	M	H/L	1	AB + itra + Pred + HAART (O)	S	Nieto et al. [35]
Venezuela/Spain, 2017	23	M	H/L	1	Lip AB + Ig + Dex + itra	S	Gómez-Espejo et al. [36]
Guyana/United States of America, 2017	49	M	H/L	1	Lip AB + HAART + IVIG > anakinra + Dex + itra (O)	S	Ocon et al. [37]
Dominican Republic, 2017	46	M	H/L	1	Lip AB + itra (O) + HAART (O)	S	Loganantharaj et al. [38]
El Salvador/United States of America, 2018	48	M	H/L	1	Dex + Lip AB + itra (O) + HAART (O)	S	Asanad et al. [39]
Venezuela/Japan, 2019	NA	F	H/L	1	HAART + Lip AB + itra	S	Tsuboi et al. [40]
United States of America, 2019	41, 48	M	W	2	Lip AB + itra	S/D	Jabr et al. [41]
French Guiana, 2020	30–40 (2), 40–50 (7), 50–60 (2), 60–70 (1)	N/A	H/L	12	LipAB + itra, Lip AB + steroids + Ig, Lip AB + itra + etoposide	S(10), D (2)	Nguyen et al. [42]

*Note:* S = survived; D = death; M = male; F = female; W = White; B = Black; A = Asian; Ig = immunoglobulin; Dex = dexamethasone; Pred = prednisone; O = started outpatient; Lip AB = liposomal amphotericin B; itra = itraconazole.

Abbreviations: AB = amphotericin B; ABD = amphotericin B deoxycholate; ABLC = amphotericin B lipid complex; AI = American Indian; H/L = Hispanic/Latino; N/A = not available; PI = Pacific Islander.

## Data Availability

The raw/processed data used to write up this case report cannot be shared at this time due to legal/ethical reasons including protected patient information/patient identifiers.
